# Tactile Stimulation to Stimulate Spontaneous Breathing during Stabilization of Preterm Infants at Birth: A Retrospective Analysis

**DOI:** 10.3389/fped.2017.00061

**Published:** 2017-04-03

**Authors:** Janneke Dekker, Tessa Martherus, Sophie J. E. Cramer, Henriette A. van Zanten, Stuart B. Hooper, Arjan B. te Pas

**Affiliations:** ^1^Department of Pediatrics, Leiden University Medical Center, Leiden, Netherlands; ^2^Department of Medical Engineering, Delft University of Technology, Delft, Netherlands; ^3^The Ritchie Center, MIMR-PHI Institute of Medical Research, Melbourne, VIC, Australia

**Keywords:** preterm, breathing, tactile, stimulation, birth, stabilization

## Abstract

**Background and aims:**

Tactile maneuvers to stimulate breathing in preterm infants are recommended during the initial assessment at birth, but it is not known how often and how this is applied. We evaluated the occurrence and patterns of tactile stimulation during stabilization of preterm infants at birth.

**Methods:**

Recordings of physiological parameters and videos of infants <32 weeks gestational age were retrospectively analyzed. Details of tactile stimulation during the first 7 min after birth (timing, duration, type, and indication) were noted.

**Results:**

Stimulation was performed in 164/245 (67%) infants. The median (IQR) GA was 28 6/7 (27 2/7–30 1/7) weeks, birth weight 1,153 (880–1,385) g, Apgar score at 5 min was 8 (7–9), 140/245 (57%) infants were born after cesarean section, and 134/245 (55%) were male. There were no significant differences between the stimulated and the non-stimulated infants with regard to basic characteristics. In the stimulated infants, the first episode of stimulation was given at a median (IQR) of 114 (73–182) s after birth. Stimulation was repeated 3 (1–5) times, with a median (IQR) duration of 8 (4–16) s and a total duration of 32 (15–64) s. Modes of stimulation were: rubbing (68%) or flicking (2%) the soles of the feet, rubbing the back (12%), a combination (9%), or other (8%). In 67% of the stimulation episodes, a clear indication was noted (25% bradycardia, 57% apnea, 48% hypoxemia, 43% combination) and an effect was observed in 18% of these indicated stimulation episodes. A total effect of all stimulation episodes per infant remains unclear, but infants who did not receive stimulation were more often intubated in the delivery room (14/79 (18%) vs 12/164 (7%), *p* < 0.05).

**Conclusion:**

There was a large variation in the use of tactile stimulation in preterm infants during stabilization at birth. In most cases, there was an indication for stimulation, but only in a small proportion an effect could be observed.

## Introduction and Rationale

Most preterm infants initiate breathing after birth, but their respiratory drive is weak and often insufficient (1–5). However, in the last decade, the focus of respiratory care in the delivery room has shifted from intubation and mechanical ventilation toward non-invasive ventilation and supporting spontaneous breathing (2, 6). Both local and international resuscitation guidelines recommend to assess respiratory effort, and if necessary, stimulate and support spontaneous breathing (7–9).

Tactile stimulation (warming, drying, and rubbing the back or the soles of the feet) has been recommended in the guidelines to stimulate spontaneous breathing (7–9). Although this is now a commonly accepted intervention, the effect remains unclear. Experimental studies have shown a positive effect of tactile stimulation on spontaneous breathing at birth (10, 11), but there is very little human data demonstrating the effect of stimulation, especially in preterm infants.

Although most interventions in the delivery room have been evaluated (12–17), frequency and method of tactile stimulation have not been evaluated objectively (12–17). Besides the description of several studies that neonatal caregivers often defer from the resuscitation guidelines (12–17), the use of tactile stimulation might be influenced by the current practice that preterm infants are not dried but placed in a plastic wrap to prevent hypothermia (18, 19).

We, therefore, evaluated the occurrence and methods of tactile stimulation of preterm infants directly after birth.

## Materials and Methods

In a retrospective study, we reviewed all neonatal stabilization procedures at birth of infants with a gestational age of <32 weeks from January 2007 until June 2016 in the Leiden University Medical Center (LUMC). In this study, recordings of videos and physiological parameters of neonatal resuscitation in the delivery room were used. The recording of videos and physiological parameters of resuscitation in the delivery room for auditing is standard of care at the LUMC.

Videos and respiratory function monitoring (RFM), including heart rate, oxygen saturation, and fraction of inspired oxygen were recorded as soon as the infants’ shoulder was out during delivery. Respiratory parameters were recorded with either a Florian RFM (Acutronic Medical Systems AG, Hirzel, Switzerland), using a hot wire anemometer, or a New Life Box (Applied Biosignals, Weener, Germany) connected to a MRT-A RFM (Applied Biosignals, Weener, Germany), using a variable orifice pneumometer (Avea Varflex Flow transducer) (Carefusion, Yorba Linda, CA, USA). Oxygen saturation and heart rate were recorded using a Masimo SET pulse oximeter (Masimo Radical, Masimo Corporation, Irvine, CA, USA). The pulse oximetry probe was placed around the infant’s right wrist. In case the Florian RFM was used, gas flow, pressures given, tidal volume, oxygen saturation, heart rate, and breathing signals were digitized using the Spectra physiological software (Grove Medical Limited, Hampton, UK). Polybench software (Applied Biosignals, Weener, Germany) was used when making use of the New Life Box.

The videos and physiological parameters were independently reviewed and analyzed by two researchers involved in the study (Janneke Dekker and Tessa Martherus). In case of doubt, consensus was achieved with the help of a third researcher (Arjan B. te Pas), to guarantee objectivity of the analyzing process. The logging of the occurrence and methods of tactile stimulation was started after the infant was dried or put in the plastic wrap, the cap was put on, and the pulse oximeter and CPAP/Neopuff mask were placed.

For all included infants, we collected the following patient characteristics: gestational age at birth, birth weight, gender, mode of delivery, Apgar score at 5 min after birth, antenatal corticosteroids, and intubation in the delivery room.

The main variable of interest was the occurrence of tactile stimulation of the neonate in the first 7 min after birth, as the majority of infants are being prepared for transport to the NICU after 7 min. We also noted the frequency and duration of tactile stimulation per infant, the time points and the method of stimulation (rubbing the back, rubbing the soles of the feet, flicking the soles of the feet, other). If stimulation was performed, we noted whether there was an indication for stimulation based on clinical signs such as apnea/irregular breathing, wherefore the infant needed positive pressure ventilation, bradycardia (a heart rate <100 bpm), or hypoxia (oxygen saturation below the recommended target range described by Wyllie et al. (20)). In case of a clear clinical indication, we also noted the effect (recovery of heart rate >100 bpm and/or regaining breathing/increased breathing effort). When tactile stimulation was not performed, we noted whether stimulation could have been indicated.

After all data of individual stimulation episodes was logged, a categorical scheme was drafted by two of the researchers (Janneke Dekker and Tessa Martherus) in which patterns of stimulation were explicated. After this, all videos were reviewed and coded to one of the patterns.

This study was conducted according to the principles of the Declaration of Helsinki and in accordance with the Medical Research Involving Human Subjects Act (WMO). In the Netherlands, no ethical approval is required for anonymized studies with medical charts, and patient data that are used for daily care. The Research Ethics Committee issued a statement of no objection.

### Statistical Analysis

Results are presented as mean ± SD for normally distributed values or medians (IQR) for non-normally distributed values. The demographical data of stimulated infants were compared with non-stimulated infants using Student’s *t* test for parametric variables, the Mann–Whitney *u* test for non-parametric comparisons, and the *X*^2^ test for categorical variables. *p* < 0.05 was considered statistically significant, reported *p* values are two sided. Data analysis was performed using IBM SPSS Statistics version 23 (IBM Software, New York, NY, USA, 2012). Missing values were excluded case wise from the analysis if they represented less than 5% of total values. Otherwise, multiple imputation was used. The remaining missing values were excluded case wise.

## Results

**Figure 1:**
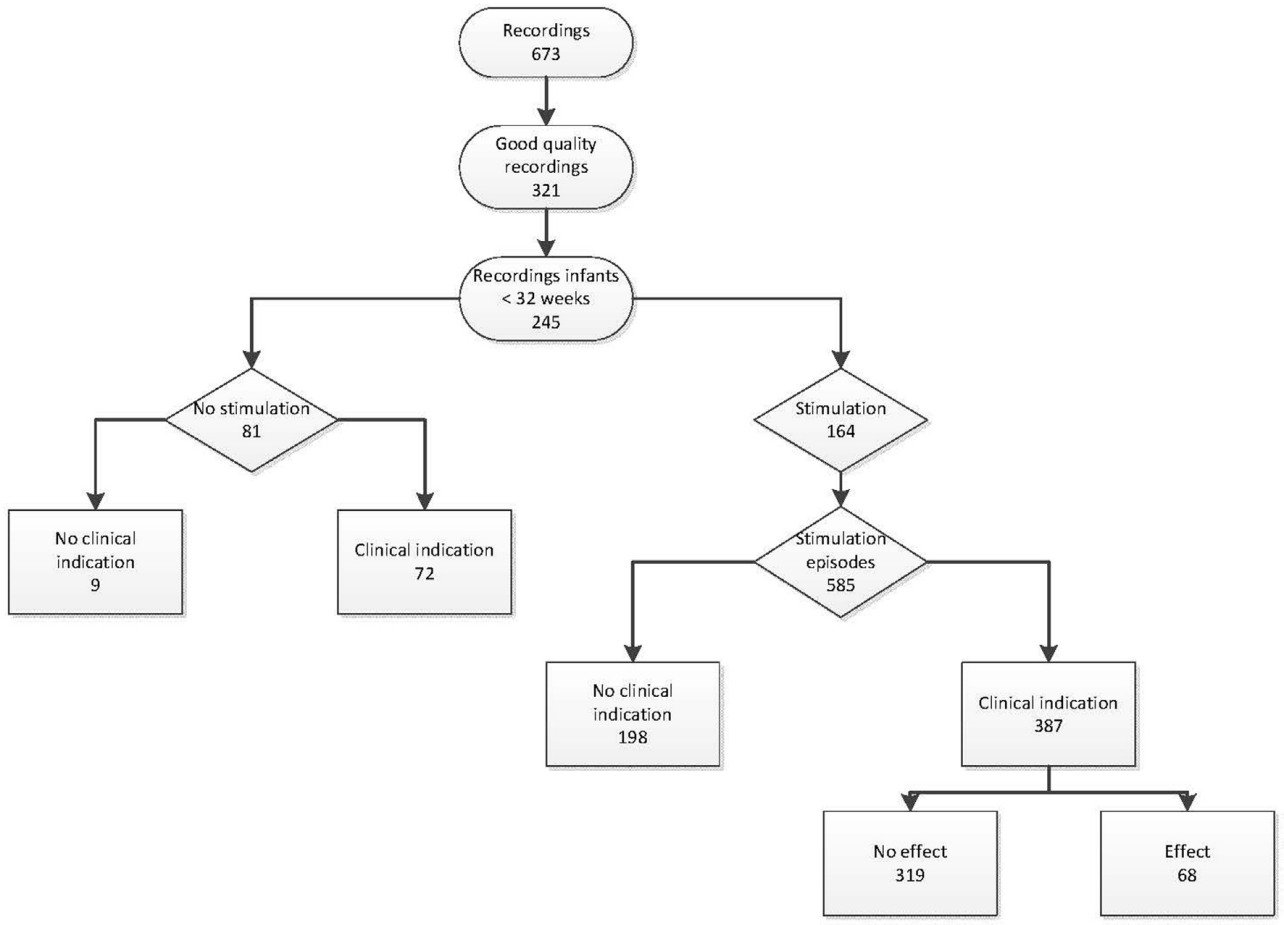
**Flowchart**.

A total of 673 infants were recorded, of which 321 recordings were complete and of good quality. From these, 245 recordings included stabilization at birth of infants born with a gestational age <32 weeks and were included in the analysis (Figure 1). The median (IQR) GA was 28 6/7 (27 2/7–30 1/7) weeks, birth weight was 1,153 (880–1,385) g, Apgar score at 5 min was 8 (7–9), 140/245 (57%) infants were born after cesarean section, 153/245 (62%) received antenatal corticosteroids, and 134/245 (55%) infants were male.

Tactile stimulation was performed in 164/245 (67%) infants. GA, birth weight, gender, mode of delivery, antenatal corticosteroids, and Apgar did not differ significantly between stimulated and non-stimulated infants (Table 1). The first moment of stimulation started at median (IQR) 114 (73–182) s after birth. Each stimulation episode had a median (IQR) duration of 8 (4–16) s. The total time of stimulation was 32 (15–64) s. Four different stimulation patterns could be identified and categorized, based on information about indication and repetitiveness of stimulation (Table 2).

**Table 1:** **Demographical data**.

	Stimulated infants	Non-stimulated infants	*p*-Value
		
	*N* = 164	*N* = 81	
Gestational age (weeks)^a^	29 0/7 (27 3/7–30 2/7)	28 4/7 (26 6/7–30 0/7)	0.298
Gender (% male)^b^	94/163 (58%)	40/80 (50%)	0.419
Birth weight^a^	1,165 (875–1,418)	1,121 (880–1,363)	0.543
Mode of delivery (% cesarean section)^b^	93/164 (57%)	47/81 (58%)	0.893
Antenatal corticosteroids^b^	106/149 (71%)	47/70 (67%)	0.636
Apgar score at 5 min after birth^a^	8 (6–9)	8 (7–9)	0.669

*Data are presented as median (IQR) for non-parametric data (^a^) and *n* (%) for categorical data (^b^)*.

**Table 2:** **Stimulation types**.

Stimulation type	Incidence *n* (%)
Indicated repetitive stimulation	29/164 (18)
Non-indicated repetitive stimulation	2/164 (1)
Indicated non-repetitive stimulation	101/164 (62)
Non-indicated non-repetitive	32/164 (20)

In the 164 infants that received stimulation, a total of 585 stimulation episodes were observed with median (IQR) 3 (1–5) stimulations per infant. Stimulation was performed most often by rubbing the soles of the feet (Table 3). In 387/578 (67%) stimulation episodes, a clear clinical indication for stimulation could be observed, which were bradycardia (25%), apnea/irregular breathing (57%), hypoxia (48%), or a combination of these (43%). An effect could be observed in 68/387 (18%) of these stimulation episodes.

**Table 3:** **Methods of stimulation**.

Method of stimulation	Incidence *n* (%)
Rubbing the soles of the feet	400/585 (68)
Rubbing the back	70/585 (12)
Flicking the soles of the feet	10/585 (2)
Combination of abovementioned methods	55/585 (9)
Other (vigorous rubbing, drying, rubbing extended to the lower extremities)	49/585 (8)

Although 81/245 (33%) infants received no stimulation during resuscitation, a clinical indication during the resuscitation could be observed in 72/81 (89%) infants (bradycardia 67%, apnea 70%, hypoxia 70%, or a combination of these 69%).

A total of 26/243 (11%) infants were intubated in the delivery room. The incidence of intubation in the delivery room was significantly higher in infants who received no stimulation compared to the stimulated infants [14/79 (18%) vs 12/164 (7%), *p* < 0.05]. When comparing infants with a clinical indication for stimulation, the incidence of intubations was also significantly higher in non-stimulated infants [14/70 (20%) vs 12/130 (9%), *p* < 0.05].

## Discussion

This is the first study where recordings of video and physiological parameters were used to review the use of tactile stimulation in preterm infants at birth. In the majority of infants, tactile stimulation was applied during stabilization at birth. In most cases, there was a clinical indication for stimulation, although still a proportion of infants did not receive stimulation while this was indicated. In addition, the starting time, duration, and method of tactile stimulation varied between infants. Although we could not observe a clear direct effect of each stimulation episode in infants where stimulation was indicated, a total effect of all stimulation periods in an infant remains unclear. Indeed, when stimulation was indicated, infants who did not receive stimulation were more often intubated.

Tactile stimulation is suggested in international guidelines (7–9), but there are no clear recommendations on indication for tactile stimulation, timing, and method of tactile stimulation. This is probably due to the scarcity in data how tactile stimulation can be best used during stabilization of preterm infants at birth. The lack of a definition in the guidelines on timing and method of tactile stimulation probably explains the large variation we observed in practice.

Although the effect of tactile stimulation as single intervention has not been described in human preterm infants at birth, this has been demonstrated in animals. Faridy et al. (10) described that maternal rats perform rolling, licking, biting, and pushing of the newborn rat to stimulate its breathing. When newborn rats were removed from their mother directly after birth, they developed respiratory distress (10). In apneic term, lambs spontaneous breathing commenced when they were stimulated with both mechanical and electrical stimuli (11).

Breathing is initiated by the respiratory center in the medulla by different stimuli including hypoxia and hypercapnia (21, 22). On the other hand, the respiratory center can be depressed by the level of adenosine present at birth, which may vary widely under different conditions such as mode of delivery and timing of cord clamping (23–25). Breathing effort can be increased by changing the state of arousal of the infant by the use of tactile stimulation. The response on arousal may vary according to the location of the nerves that are stimulated (26). Ioffe et al. (27) conducted a study in fetal lambs to test the respiratory response to somatic stimuli and found that the sleep state (NREM, REM, awake) changed when stimuli were given. According to this study, the respiratory response was greatest when fetal lambs were in REM sleep or awake at the end of stimulation (27, 28).

However, the effect of tactile stimulation on breathing by means of arousal is not clear as infants are exposed to other stimuli at birth, which might change their arousal state, such as light, cold, and sound. In addition, most preterm infants receive respiratory support, which could also stimulate breathing effort (29). In contrast, it could be possible that in the effort of applying tactile stimulation, the focus shifts away from the other interventions during stabilization. In addition, vigorous stimulation could potentially lead to displacement of the mask.

In contrast to tactile stimulation at birth, stimulation to counteract apnea of prematurity after admittance to the NICU has been well studied in human infants, with stimulation performed in different ways. In the study of Kattwinkel et al. (30), when extremities were rubbed repetitively as method of tactile stimulation, the frequency of apnea was significantly reduced during repetitive stimulation and the 2 h after the repetitive stimulation (30). In addition, the use of a stochastic resonance mattress is shown to reduce the incidence of apnea (31, 32). However, other methods of stimulation like the use of kinesthetic stimulation have not shown to be effective as treatment for apnea of prematurity in the NICU (33). It is assumed that underdevelopment of the respiratory center in the medulla, hypoxia, or altered sensitivity of chemoreceptors to carbon dioxide or oxygen might be the cause of apnea of prematurity (30). However, the mechanisms underlying apnea during prematurity might differ from the mechanisms of apnea at birth.

Although this study is limited by its retrospective design, objective parameters and videos could be used to observe the current practice in tactile stimulation as also the effect of stimulation. However, the decision to apply stimulation remains to the discretion of caregivers. Neither the motivation whether stimulation was performed nor the preferred methods were noted. Since we reviewed the recordings from 2007 to 2016, it is possible that the use of stimulation has changed since our practice has changed toward more support of spontaneous breathing. However, we could not observe a change along the timeline of analyzed recordings. As the video recordings were made anonymous with regard to the caregivers in charge of the stabilization procedure, we could not assess patterns in stimulation among different caregivers. Although there were no differences in characteristics between the groups, it is possible that other unmeasured variables besides stimulation influenced the intubation rate.

In summary, we observed that there was a large variability in the use of tactile stimulation during stabilization of preterm infants at birth. When stimulation was applied most often this was indicated, while there was also often an indication when no stimulation was given at all. We could not observe a direct effect of stimulation in most occasions. While there is increasing awareness that most preterm infants breathe at birth and there is more emphasis on supporting this, stimulating breathing effort will also play an important role in this. The variation observed in current practice indicates that studies are warranted on duration and type of tactile stimulation leading to a better definition in guidelines when and how stimulation should be performed.

## Author Contributions

JD and AP conceptualized the study. JD, TM, and HZ analyzed the data, and JD wrote the first draft of the manuscript. JD, SC, SH, and AP contributed to the interpretation of the data. All the authors critically reviewed and contributed to the final draft of the manuscript.

## Conflict of Interest Statement

The authors declare that the research was conducted in the absence of any commercial or financial relationships that could be construed as a potential conflict of interest.

## References

[1] Schilleman K van der Pot CJ Hooper SB Lopriore E Walther FJ te Pas AB. Evaluating manual inflations and breathing during mask ventilation in preterm infants at birth. J Pediatr (2013) 162(3):457–63. 10.1016/j.jpeds.2012.09.036 23102793

[2] van Vonderen JJ Hooper SB Hummler HD Lopriore E te Pas AB. Effects of a sustained inflation in preterm infants at birth. J Pediatr (2014) 165(5):903–8.e1. 10.1016/j.jpeds.2014.06.007 25039041

[3] O’Donnell CP Kamlin CO Davis PG Morley CJ. Crying and breathing by extremely preterm infants immediately after birth. J Pediatr (2010) 156(5):846–7. 10.1016/j.jpeds.2010.01.007 20236659

[4] Trevisanuto D Satariano I Doglioni N Criscoli G Cavallin F Gizzi C Changes over time in delivery room management of extremely low birth weight infants in Italy. Resuscitation (2014) 85(8):1072–6. 10.1016/j.resuscitation.2014.04.024 24791692

[5] SUPPORT Study Group of the Eunice Kennedy Shriver NICHD Neonatal Research Network Finer NN Carlo WA Walsh MC Rich W Gantz MG Early CPAP versus surfactant in extremely preterm infants. N Engl J Med (2010) 362(21):1970–9. 10.1056/NEJMoa0911783 20472939 PMC3071534

[6] O’Donnell CP Schmolzer GM. Resuscitation of preterm infants: delivery room interventions and their effect on outcomes. Clin Perinatol (2012) 39(4):857–69. 10.1016/j.clp.2012.09.010 23164183

[7] Wyckoff MH Aziz K Escobedo MB Kapadia VS Kattwinkel J Perlman JM Part 13: neonatal resuscitation: 2015 American Heart Association guidelines update for cardiopulmonary resuscitation and emergency cardiovascular care. Circulation (2015) 132(18 Suppl 2):S543–60. 10.1161/CIR.0000000000000267 26473001

[8] Wyllie J Perlman JM Kattwinkel J Wyckoff MH Aziz K Guinsburg R Part 7: neonatal resuscitation: 2015 international consensus on cardiopulmonary resuscitation and emergency cardiovascular care science with treatment recommendations. Resuscitation (2015) 95:e169–201. 10.1016/j.resuscitation.2015.07.045 26477424

[9] Lee AC Cousens S Wall SN Niermeyer S Darmstadt GL Carlo WA Neonatal resuscitation and immediate newborn assessment and stimulation for the prevention of neonatal deaths: a systematic review, meta-analysis and Delphi estimation of mortality effect. BMC Public Health (2011) 11(Suppl 3):S12. 10.1186/1471-2458-11-S3-S12 21501429 PMC3231885

[10] Faridy EE. Instinctive resuscitation of the newborn rat. Respir Physiol (1983) 51(1):1–19. 10.1016/0034-5687(83)90098-1 6836195

[11] Scarpelli EM Condorelli S Cosmi EV. Cutaneous stimulation and generation of breathing in the fetus. Pediatr Res (1977) 11(1 Pt 1):24–8. 10.1203/00006450-197711010-00007 556651

[12] Mann C Ward C Grubb M Hayes-Gill B Crowe J Marlow N Marked variation in newborn resuscitation practice: a national survey in the UK. Resuscitation (2012) 83(5):607–11. 10.1016/j.resuscitation.2012.01.002 22245743 PMC3350052

[13] O’Donnell CP Davis PG Morley CJ. Neonatal resuscitation: review of ventilation equipment and survey of practice in Australia and New Zealand. J Paediatr Child Health (2004) 40(4):208–12. 10.1111/j.1440-1754.2004.00339.x 15009551

[14] Iriondo M Thio M Buron E Salguero E Aguayo J Vento M A survey of neonatal resuscitation in Spain: gaps between guidelines and practice. Acta Paediatr (2009) 98(5):786–91. 10.1111/j.1651-2227.2009.01233.x 19243354

[15] Schilleman K Siew ML Lopriore E Morley CJ Walther FJ Te Pas AB. Auditing resuscitation of preterm infants at birth by recording video and physiological parameters. Resuscitation (2012) 83(9):1135–9. 10.1016/j.resuscitation.2012.01.036 22322286

[16] Singh Y Oddie S Marked variation in delivery room management in very preterm infants. Resuscitation (2013) 84(11):1558–61. 10.1016/j.resuscitation.2013.06.026 23948446 PMC3828483

[17] Konstantelos D Dinger J Ifflaender S Rudiger M. Analyzing video recorded support of postnatal transition in preterm infants following a c-section. BMC Pregnancy Childbirth (2016) 16:246. 10.1186/s12884-016-1045-2 27561701 PMC5000427

[18] Rohana J Khairina W Boo NY Shareena I. Reducing hypothermia in preterm infants with polyethylene wrap. Pediatr Int (2011) 53(4):468–74. 10.1111/j.1442-200X.2010.03295.x 21105964

[19] Morley C New Australian neonatal resuscitation guidelines. J Paediatr Child Health (2007) 43(1–2):6–8. 10.1111/j.1440-1754.2007.01021.x 17207048

[20] Wyllie J Perlman JM Kattwinkel J Atkins DL Chameides L Goldsmith JP Part 11: Neonatal resuscitation: 2010 International Consensus on Cardiopulmonary Resuscitation and Emergency Cardiovascular Care Science with Treatment Recommendations. Resuscitation (2010) 81(Suppl 1):e260–87. 10.1016/j.resuscitation.2010.08.029 20956039

[21] Harding R Fetal pulmonary development: the role of respiratory movements. Equine Vet J Suppl (1997) 24:32–9. 10.1111/j.2042-3306.1997.tb05076.x 9355800

[22] Hooper SB Harding R. Fetal lung liquid: a major determinant of the growth and functional development of the fetal lung. Clin Exp Pharmacol Physiol (1995) 22(4):235–47. 10.1111/j.1440-1681.1995.tb01988.x 7671435

[23] Irestedt L Dahlin I Hertzberg T Sollevi A Lagercrantz H. Adenosine concentration in umbilical cord blood of newborn infants after vaginal delivery and cesarean section. Pediatr Res (1989) 26(2):106–8. 10.1203/00006450-198908000-00007 2771515

[24] Thorburn GD The placenta and the control of fetal breathing movements. Reprod Fertil Dev (1995) 7(3):577–94. 10.1071/RD9950577 8606971

[25] Crossley KJ Nicol MB Hirst JJ Walker DW Thorburn GD. Suppression of arousal by progesterone in fetal sheep. Reprod Fertil Dev (1997) 9(8):767–73. 10.1071/R97074 9733059

[26] Marayong P Mostoufi MS. Foot vibrotactile device for central apnea interruption in premature infants. Stud Health Technol Inform (2009) 142:180–2. 10.3233/978-1-58603-964-6-180 19377144

[27] Ioffe S Jansen AH Russell BJ Chernick V. Respiratory response to somatic stimulation in fetal lambs during sleep and wakefulness. Pflugers Arch (1980) 388(2):143–8. 10.1007/BF00584120 7192851

[28] Dawes GS Fox HE Leduc BM Liggins GC Richards RT. Respiratory movements and rapid eye movement sleep in the foetal lamb. J Physiol (1972) 220(1):119–43. 10.1113/jphysiol.1972.sp009698 4333826 PMC1331693

[29] Schmolzer GM Kumar M Pichler G Aziz K O’Reilly M Cheung PY. Non-invasive versus invasive respiratory support in preterm infants at birth: systematic review and meta-analysis. BMJ (2013) 347:f5980. 10.1136/bmj.f5980 24136633 PMC3805496

[30] Kattwinkel J Nearman HS Fanaroff AA Katona PG Klaus MH. Apnea of prematurity. Comparative therapeutic effects of cutaneous stimulation and nasal continuous positive airway pressure. J Pediatr (1975) 86(4):588–92. 10.1016/S0022-3476(75)80158-2 1092821

[31] Bloch-Salisbury E Indic P Bednarek F Paydarfar D. Stabilizing immature breathing patterns of preterm infants using stochastic mechanosensory stimulation. J Appl Physiol (1985) (2009) 107(4):1017–27. 10.1152/japplphysiol.00058.2009 19608934 PMC2763836

[32] Smith VC Kelty-Stephen D Qureshi Ahmad M Mao W Cakert K Osborne J Stochastic resonance effects on apnea, bradycardia, and oxygenation: a randomized controlled trial. Pediatrics (2015) 136(6):e1561–8. 10.1542/peds.2015-1334 26598451 PMC4657600

[33] Osborn DA Henderson-Smart DJ Kinesthetic stimulation for treating apnea in preterm infants. Cochrane Database Syst Rev (2000) 2:CD000499 10.1002/14651858.CD000499 PMC7078912 10796212

